# Restorative Storytelling for Migrants’ and Refugees’ Mental Wellbeing and Community Empowerment in Canada: A Scoping Review

**DOI:** 10.1177/13634615261459629

**Published:** 2026-06-30

**Authors:** Joseph Adu, Rade Zinaic, Priscilla N. Boakye, Yemisi Onilude, Raliat A. Owolabi, Esther Olukayode, Tania Correa, Josephine Pui-Hing Wong

**Affiliations:** 1 Daphne Cockwell School of Nursing, 7984 Toronto Metropolitan University, Toronto, Canada

**Keywords:** restorative storytelling, mental health, immigrant communities, community theatre, Canada

## Abstract

Storytelling is a widely used method in community-based research. We conducted a scoping review to explore restorative storytelling and community theatre as a tool to support mental health literacy and wellness as well as equity among immigrant communities. Our review followed the methodological framework espoused by Arksey and O’Malley and modified by Levac et al. All 13 peer-reviewed studies that met the inclusion criteria were conducted in Canada. Twelve of the included studies employed qualitative methods, with only one using a quantitative approach. The included studies involved diverse immigrants living in communities across Canada. We identified five themes that spoke to the objective of the review: (1) stories of social marginalization and barriers; (2) increased awareness and understanding through stories; (3) storytelling as a space for transformation and healing; (4) connectedness and sharing through stories; and (5) storytelling promoting empowerment and resilience. Language barriers alongside race were identified as major hurdles encountered by some immigrants. Digital storytelling was seen by some immigrants as a process that provided the opportunity to reflect and reminisce on life, which fostered understanding and (re)connection with self and, in so doing, improved emotional wellbeing. Given the increasing mental health disparities in immigrant and racialized communities in Canada, efforts toward improving the mental wellbeing of immigrants through restorative storytelling and community theatre warrant further investigations.

Storytelling is a widely used method in community-based research (Nordin et al., 2023). Narratives and the act of storytelling help people make sense of their lived experiences and contribute to the dissemination of information. Prior research has shown that storytelling can provide marginalized communities with a means through which to share their stories and empower themselves (Caxaj, 2015; Christensen, 2012; Datta, 2018; Williams et al., 2003). Stories also help us to make sense of our experiences and environment and contribute to education and knowledge sharing (Bietti et al., 2019). According to Rouhani (2019), storytelling offers a platform through which the voices of marginalized groups can be heard on important issues. This is an empowering practice for many marginalized groups (e.g., people with intellectual disabilities) (Saridaki & Meimaris, 2018).

Recent studies have begun to explore what is known as ‘restorative storytelling,’ which foregrounds experiences of recovery and hope over the psychosocial burdens of trying circumstances (Dahmen, 2019; Fitzgerald et al., 2020b; Tenore, 2014). Thus, restorative storytelling is said to truthfully convey the emotional and psychological experiences of people in adverse situations. Such stories also emphasize resilience, compassion for others, and/or optimism toward recovery (Fitzgerald et al., 2020a; Tenore, 2014).

In line with current theories, restorative storytelling possesses two central aspects: (1) it is strengths based; and (2) it works toward meaningful progression and away from trauma and suffering (Fitzgerald et al., 2020a, 2020b; Park et al., 2004). By meaningful progression, we mean the dynamic role of restorative storytelling in facilitating a post-trauma recovery process rather than merely describing the traumatic and/or challenging event. Although restorative storytelling may include obstacles and challenges, the overall trajectory is positive (Fitzgerald et al., 2020a). Underpinned by a strengths-based approach, facilitators of restorative storytelling focus on individual and community characteristics such as optimism, gratitude, and determination that enable people to endure difficult times (Park et al., 2004); in short, restorative storytelling approaches emphasize themes of strength and psychological resilience (Fitzgerald et al., 2020b; Tenore, 2014). The act of restorative storytelling mitigates the impact of stories that may trigger intense emotions in audiences. For instance, consuming a refugee's story of loss or war-related violence can evoke feelings of sadness. However, the resilience often portrayed in restorative storytelling provides hopeful and positive counterpoints to the adverse events, encouraging readers and listeners to experience more positive emotions in response to the story and greater empathy toward the characters. Previous research suggests that restorative storytelling can evoke more positive emotions (e.g., happiness) than non-restorative approaches (Paravati et al., 2022). This positive emotional response fosters a deeper understanding and connection with the characters’ experiences. In that regard, restorative storytelling is a transformative healing exercise geared toward repairing harm and cultivating a sense of empowerment, two aims that are mainstays of restorative justice.

Restorative storytelling is a modality that helps achieve restorative justice (Hajdu, 2019). Restorative justice is an approach to justice that focuses on repairing harm by allowing those who have been harmed, and those responsible for the harm, to communicate and address their needs following the wrongdoing (Canadian Intergovernmental Conference Secretariat, 2018). Restorative justice approaches identify and explore systemic injustice as the major contributing factor reinforcing and sustaining harm against immigrants and marginalized populations and, as such, propose a transformative and co-creative progression away from harm. The pathways involved in restorative storytelling's collaborative creative process allow for the centering of impacted voices and the building of self-determination and empowerment among those harmed within systems of oppression (Friskie, 2020). Immigrants herein include both migrants and refugees. The International Organization for Migration (IOM) describes ‘migrant’ as an umbrella term involving persons moving away across an international border or within a nation away from their usual place of residence, irrespective of the person's legal status, whether the movement is voluntary or involuntary, for at least 12 months (International Organization for Migration, 2019). Refugees, on the other hand, are persons forced to escape their home country and seek safety in another country —such individuals are unable to return to their country of origin for fear of persecution, armed conflict, or serious public disorder (International Organization for Migration, 2019).

Despite research showing that some immigrants have better physical and mental health upon arrival to Canada, disparities across several social determinants of health impact the psychological wellbeing of newcomers, refugees, and immigrants (Hynie, 2018; Lane et al., 2018; Salami et al., 2022). The process of immigrating to another country is very challenging and stressful for immigrant children and families and affects their mental wellbeing (Beiser & Hou, 2017). Accordingly, Lu and Ng (2019) state that it is crucial to focus on immigrant health given the growth of the immigrant population and the numerous challenges and health disparities they encounter. Ensuring the physical and mental wellbeing of immigrants and refugees not only benefits them but also ensures the unity of the broader community (Vang et al., 2017).

*This scoping review aims to explore the role of restorative storytelling as a tool for racial justice, community empowerment, and mental health promotion in immigrant communities*. In light of the numerous injustices perpetrated against marginalized communities, it is crucial to address how the epistemic silencing of marginalized voices upholds colonial knowledge. Restorative storytelling is, therefore, essential for empowerment, promoting epistemic justice, and restoring a sense of agency to these communities (Nordin et al., 2023). The paucity of literature on how immigrant communities in Canada utilize restorative storytelling for knowledge exchange and improved mental wellbeing underscores the necessity of this project. The outcomes of this scoping review will have significant implications for research, practice, and policy.

## Methods

Our scoping review is guided by the five-stage methodological framework of Arksey and O’Malley (2005) and the modifications proposed by Levac et al. (2010). We chose these scoping review methodologies over other approaches because it allows researchers to survey topics they have only cursory knowledge of and ensures methodological consistency. Our framework thus comprises six steps, the first five from Arksey and O’Malley and the sixth one from Levac, Colquhoun, and O’Brien: (1) identify the research question; (2) identify suitable studies; (3) select studies; (4) extract data; (5) collate data and report the results; and (6) consultation. Consultation adds methodological rigor and should be considered a required component for scoping reviews. Finally, we employed the PRISMA-ScR checklist to confirm the transparency and possible reproducibility of the review process by other researchers.

An integral part of our methodological approach, and the context in which we deploy the above approaches, is the practice of co-creative mutual mentorship and skill acquisition. This equitable and co-participatory practice is led by a senior researcher and is aimed at building research capacity for a multidisciplinary research portfolio titled Immigrant Health and Wellbeing, part of the Bridging Divides migration research program. This portfolio focuses on various approaches to immigrant health in Canada across nine subprojects exploring topics such as the impact of community gardens on immigrant mental health and the use of translation apps in Long-Term Care (LTC) homes. Collaborative mentorship is a research practice deployed in many of these subprojects and involves fellows learning the rudiments of scoping review practices and paying them forward to other trainees. Our collaborative and dialogical approach had every member of the research team partake in formulating research questions and keywords, conducting search strategies, preparing results for deduplication, engaging abstract screening and data extraction, and cross-vetting each other’s contributions during the writing process for data validation. The entire process was informed by a consensus-based research question with reference to Arksey and O’Malley's framework:

What do we know about restorative storytelling and community theatre as a tool for racial justice, community empowerment, and mental health promotion in immigrant communities?

### Search Strategy and Study Selection

We identified and searched five academic databases for relevant peer-reviewed studies that report on primary sources of evidence in Canada from 2013 to 2023. The databases included: Scholars Portal, Sociological Abstracts, Medline PubMed, CINAHL, and Google Scholar. A senior scientist on our team assisted in modifying our search strategy and terms to retrieve germane studies for our review. We combined three or four sets of keywords relevant to the research question for search engine data retrieval. Our initial sets of keywords were “immigrant storytelling,” “mental wellbeing,” and “Canada.” The second sets were “restorative storytelling,” “immigrant,” “mental wellbeing,” and “Canada.” The third set included “restorative storytelling,” “emotional wellbeing,” “immigrant,” “refugee,” and “Canada.” The fourth set involved “applied theatre,” “racial equity,” “emotional wellbeing,” and “Canada.” These search terms were combined with applicable Boolean search operators (OR and AND) to broaden or narrow the search results. “Immigrants” herein include people who are temporary residents or landed immigrants or permanent residents of Canada and are not born in the country.

All articles retrieved from the various searches were saved in RIS files and imported to a reference management software program called Zotero for deduplication and further assessment based on our study inclusion criteria. We focused on: (1) restorative storytelling and immigrants’ mental health; (2) applied theatre, racial equity, and mental health; (3) peer-reviewed journal articles from 2013 to 2023; and (4) English-language basic searches of full-text publications in Canada. In all stages of the review process, we excluded all systematic reviews, gray literature publications, non-English language publications, and peer-reviewed articles in Canada before 2013.

Google Scholar required a different approach for data retrieval but the same search terms and timeframe applied in our other databases. A team co-lead was tasked to search the first 10 Google Scholar results pages and record them in a search strategy table with links to pertinent articles under every search entry. The results were then vetted by the other team co-lead to ensure precision and transparency in the article retrieval process.

A total of 1,742 articles were retrieved from the five databases. With the support of Zotero software, we accounted for 615 duplicates (n = 615), which left us with 1,127 articles. Given the quantum of studies to screen, we used the Zotero search bar to retrieve germane peer-reviewed articles that align with the focus of the study by using the following search terms: “stories,” “refugee,” “immigrants,” “mental health,” “theatre of the oppressed,” “theatre,” “community theatre,” and “Canada.” This approach retrieved 40 articles from the four databases in addition to 39 peer-reviewed articles selected from Google Scholar, which left the team with 79 articles for title and abstract screening. The 79 articles were shared among five team members for this task with reference to the inclusion and exclusion criteria of the review. Members of the team agreed to give reasons for including or excluding articles allotted to them. The titles and abstracts were vetted by all members of the team and all conflicts were resolved by consensus. After this process, 65 studies were excluded because they were review articles, were situated outside of Canada, did not discuss restorative storytelling and/or mental health-related issues, and/or were published outside of our timeframe. Fourteen studies met the inclusion criteria for full-text review. Two out of the 14 studies were excluded after full-text review for the following reasons: one study was gray literature and the other a primary study conducted in Australia. This left us with 12 articles for data extraction. The research team consented to mining the bibliographies of some systematic and scoping reviews we had archived to augment the included studies. Three studies that aligned with the focus of our study were screened for consideration. Three members of the research team perused these articles and one study was added to the eligible number of studies. Hence, 13 studies were included in the final analysis of this scoping review.

### Data Extraction

The study team met to discuss the data extraction exercise in connection with the review objective and assigned the articles to be extracted among five members of the team. The team lead set up a Google folder where all members had access to the extracted data. The data extraction was executed under the following headings: study reference/location, study purpose, study design, sample, and results. To ensure the data quality and integrity of the study results, different team members were assigned as second reviewers to verify the extracted data by their colleagues. The paired team members met and reconciled discrepancies observed in the extracted data. All team members also took turns to review the extracted data and thoroughly discussed the section in a two-hour team meeting before the commencement of final data analysis.

Using the extracted data, the team drew on Braun and Clarke's (2006) framework to perform thematic content analysis. Five team members participated in reading through the initial data followed by open coding and inductive searches for succinct labels or codes. The coded text was distilled to subcategories and categories. Using MS Word to compile the various codes, all five team members identified preliminary themes. The themes were reviewed and clarified by the co-leads of the research team. All team members jointly reviewed and validated the data analysis before the final write-up of the results.

**Figure 1.**
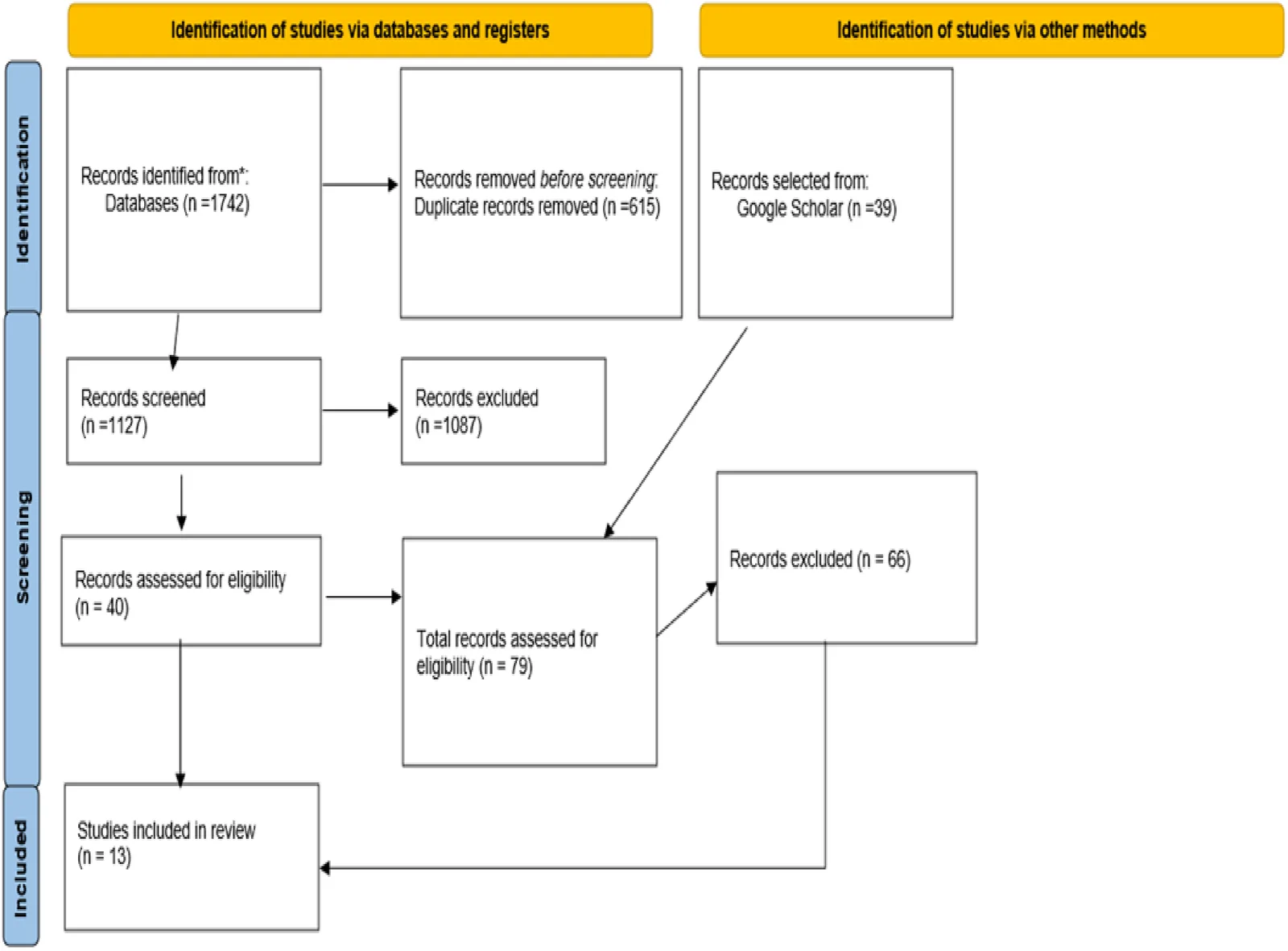
Flow chart.

## Results

### Diverse Creative Forms of Storytelling

Diverse and creative forms of storytelling were identified across many studies in this review (Brigham et al., 2018; Brushwood Rose & Granger, 2013; Chai, 2021; Epp, 2013; Hausknecht et al., 2019; Middleton et al., 2021; Schroeter, 2013; Taylor et al., 2022). Varied arts-based narrative therapy was reported to foster intergenerational communication and understanding between grandparents and young people and even change parents’ perspectives about homosexuality in many immigrant communities (Wong & Poon, 2013). Storytelling practiced through participant-generated photos fostered self-reflection and produced knowledge centered on the participants themselves (Brigham et al., 2018; Chai, 2021). For example, photo stories helped immigrant and refugee women display their settlement experiences in Canada and imbue their complex nature with meaning (Brigham et al., 2018; Brushwood Rose & Granger, 2013; Chai, 2021). Narrating experiences through photographs and stories also aided refugee women to raise awareness about the intricacies and traumas of their migration journeys (Brigham et al., 2018; Hausknecht et al., 2019). In another study with older adults, digital storytelling was seen by participants as a process that provided an opportunity to reflect and reminisce on life, which in turn fostered understanding, connection/reconnection with the self, and emotional and psychological wellbeing (Hausknecht et al., 2019). In addition, retelling one's life stories through digital stories was shown to help change some participants’ perspectives, interpreting challenging experiences in a more positive light and promoting healing (Hausknecht et al., 2019), while Middleton et al. (2021) outlined how immigrant youth engaged in arts-based activities to organize their thoughts while feeling confident sharing stories on forced migration.

Furthermore, empowering immigrants through creative activities such as participatory photography helps them reflect on their experiences, boosting their confidence and ability to face challenges (Chai, 2021; Schroeter, 2013; Taylor et al., 2022). Participant-generated images allowed immigrants to exercise autonomy in producing vivid and authentic personal narratives (Chai, 2021). Moreover, Schroeter (2013) found that critical arts-based teaching in school created a platform for marginalized students to express their bad experiences, thus allowing immigrant students to communicate and navigate their identities, aspirations, and challenges in Canada. For example, Taylor et al. (2022) observed an improvement in participants’ formerly risky sexual behaviors after they had participated in a theatre-based youth sex education program called SExT. Participants were empowered to acknowledge the consequences of unsafe sex and the importance of safely talking about sex with friends and family members (Taylor et al., 2022).

**Table 1.** Included Study Characteristics.

Reference and location	Study aim/purpose	Study design/methods	Population and sample size	Results
Beauregard et al., 2020 Canada	The paper describes various community arts projects carried out in two different neighborhoods of Montreal (Canada) to address the present increase in intercommunity tensions and to document their influence on the social connections between people from diverse ethnocultural communities, migratory status, and generations.	Qualitative multiple case study, using community-based participatory and arts-based approach, including participant observation. Arts-based data collection methods (photography, video, and arts productions).	Three projects in two socioeconomically deprived neighborhoods of Montreal. The Montreal Museum of Fine Arts produced the arts.	Collaboration and community arts enabled participants to achieve community resilience through stories. Participants had the opportunity to transform their community through community art and social connection. Arts-based storytelling fostered a sense of unity and advanced social cohesion. Stories reflected immigrant experiences of racism and discrimination; animosity, embodied loss, and battle for survival.
Brigham et al., 2018 Canada	The study aimed to examine the learning processes of women in Nova Scotia who had recently immigrated to Canada through participatory photography.	Qualitative approach; using participatory photography as a research method.	Immigrants from diverse countries (N = 20). First project: n = 10 men and women. Second project: n = 15 young immigrant and refugee women.	Participatory photography was used in storytelling; photo images enhanced deeper understanding of immigrants’ diverse identities; personal stories facilitated a sense of unity and feeling “not alone”; participatory photography enabled participants to feel in charge of their own lives.
Chai, 2021 Canada	Aim to understand the settlement experiences and needs of immigrant women in a small Canadian town (Red Deer).	Qualitative photovoice research with participant-generated photos. In-depth semi-structured one-on-one follow-up interview.	Immigrant women of diverse (N = 32): (economic, family, refugee classes) backgrounds; aged 19–48 years from Asia, Africa, and South America; in Canada < 10 yrs; 19 with a university degree; 13 high school or less.	Participants were able to examine and interpret significant aspects of their settlement experience through the picture story. The participant-generated photographs in the research were beneficial because it is more relatable, real, and emotional. The story behind each image illustrated immigrant women's vulnerability (feeling unsafe in public spaces) and resilience (establishing positive connections with others in safe environments; using library services to improve English proficiency).
Epp, 2013 Canada	The study aimed to address intergenerational conflict and build intergenerational alliances within the Tamil community.	Qualitative study using intergenerational alliance sessions as the primary method of data collection.	Youth, parents, and grandparents in Tamil communities (N = 42).	Storytelling revealed community challenges such as depression, alcohol and drug use, youth suicide, illness, loneliness, youth dropping out of school, frustration and stress. Stories also identified community support, friends, faith in God, prayers, comfort food, and music helped participants to navigate their challenges. Storytelling through role-play allowed participants to explore intergenerational conflict resulting from cultural disparities and generational divides.
Hausknecht et al., 2019 Canada	The research project explored the possible benefits and experiences of older adults who took one of our digital storytelling courses and gain a better understanding of such areas as socioemotional aspects of the process.	Mixed-method study with qualitative digital storytelling; demographics questionnaire. Post-course surveys w/ qualitative & quantitative questions. Post-course focus group interviews w/ semi-structured questions & thematic analysis of focus group transcripts.	Female and male immigrants, n = 41; aged 55–90, mostly 60–89. 47 viewers: family, friends, participants, community members. N = 88.	Digital storytelling enhanced social interaction, collaborative learning, shared understanding, and mutual support; it enables participants to reflect on life, create emotional connection, and build relationships.
Middleton et al., 2021 Canada	The primary objective was to facilitate intercultural understanding by highlighting the voices of forced immigrant youths in the knowledge development phase of a community-based participatory action research (CBPAR) project.	The study utilized arts-based conversational interviews as part of a CBPAR project. A polyphonic vignette method was employed to portray an interpretive account of the stories told by 22 refugee and asylum-seeking youth.	The sample consisted of 22 forced immigrant youth, including refugees and asylum seekers. These youths were from countries like Syria, Nigeria, Libya, and Chad and had resettled in Canada. Most participants were Muslim, and the sample was predominantly male. The youth who shared their stories were born in Syria (n = 17), Nigeria (n = 2), Libya (n = 2), and Chad (n = 1). N = 22.	Participants used stories to constantly navigate several cultural barriers in relation to sports activities. Positive outcomes of participating in sport include improved language acquisition, relationship building, and high self-esteem. Participants were comfortable sharing their stories through drawing. The narrative presented through the sport program helps in comprehending what safe space may entail for immigrant youths. Storytelling helped immigrant youths to restore their mental health and build the resilience necessary to deal with their traumatic pasts.
Brushwood Rose & Granger, 2013 Canada	To explore the complex social and psychological factors that are both barriers and motivators to newcomer and low-income women who access community-based adult learning opportunities, including dynamics of social difference, family and community support, and previously held beliefs about education and themselves as learners.	Qualitative study. Digital storytelling as context for research and as method of data collection. Data collection methods included digital story workshops, short writing exercises, group discussions within a “story circle,” interviews. In-depth interviews.	Newcomer & low-income women. First digital storytelling workshop: (2 h/wk for 16 wks). n = 8. Languages spoken: Tamil, Bengali, Mandarin, Somali, English. Second workshop: (one full day/wk for 9 wks). n = 8. Participants were from China, Indonesia, Bangladesh, Egypt, and Afghanistan. Only some of the 16 participated in the posthumous in-depth interviews. N = 16.	Alienation, feeling of disconnection, and isolation from family and loved ones were frequent themes in the digital stories. Participants spoke about the complex and occasionally unresolved nature of their narratives through their choice of representation. Free association is therapeutic, and it enables participants to express their knowledge of uncertainty and loss. Narrating stories showed the difficulties of personal experiences and individual identity.
Rousseau et al., 2014 Canada	The objective was to evaluate the effectiveness of a school-based theater intervention program for immigrant and refugee youth in special classes, aimed at improving mental health and academic outcomes.	Cluster randomized- controlled trial. The primary outcome was measured using the Impact Supplement of the Strengths and Difficulties Questionnaire (SDQ) completed by the adolescents.	The sample comprised secondary school students assigned to special classes because of learning or behavioral problems, totaling 477 students with 140 in the control group, 157 in the theater intervention group, and 180 in the tutorship intervention group. Special classrooms in five multiethnic high schools were randomly assigned to theater intervention (n = 10), tutoring (n = 10), or control status (n = 9). N = 477.	Drama workshops conducted in high schools for recently arrived migrants offered a safe space for them to express themselves. The workshops enabled young people to translate their difficult situations into a learning experience. Activities were performed in a setting that encourages teamwork while respecting individual differences.
Schroeter, 2013 Canada	Examines the use of Theatre of the Oppressed as a critical pedagogy.	Qualitative research. Social anthropology. Case-study methodology. Ethnographic methods of data collection. Participant observation. Semi-structured interviews. Participatory action research. Arts-based research methods. Field notes.	6 Black African Canadian students with refugee backgrounds (2 female and 4 male). Three remaining students were young men born in Canada (1 white and 1 Black student from Quebec, and 1 White student from British Columbia). Age = 15–18 years. N = 9 students.	Participant performance demonstrated how the present system's design makes it challenging for students with refugee backgrounds to achieve success. Through counternarratives and counter storytelling, the racialized and oppressed communities described their experiences of injustice. The counter-narrative allows the participants to prove the misconception of Canada as a color-blind and inclusive society. The study highlighted the significance of language competency and how it might prevent someone from succeeding in a foreign nation. Critical arts-based pedagogies allowed the marginalized students to communicate the challenges they faced in school. The workshops were an avenue for students to refute and reframe the dominant narratives within their academic and social environments.
Taylor et al., 2022 Canada	The study aimed to design, implement, and evaluate “SExT: Sex Education by Theatre,” a novel, theatre-based, culturally empowering peer education intervention.	Participatory action research. Mixed-methods evaluation approach. Surveys, focus groups, peer interviews, and arts-based data collection.	19 peer educators comprising both male and female participants, ranging in age from 15 to 20. Most participants identified as persons of color, with a majority being first- or second-generation Canadians with immigrant backgrounds. The sample included diverse racial identities and a mix of religious beliefs.	Participants who had previously engaged in risky sexual behaviors reported improved self-efficacy and increased risk awareness after SExT. Peer educators reported gaining confidence to discuss STIs and testing after taking part in and observing role-plays. Mixed gender roles facilitated discussion about the use of contraceptives and gave participants the courage to request safer sex. A significant number of participants stated that they used sexual health services in the months after the program. Young women felt empowered knowing their sexual rights and being able to use their power effectively.
Valenzuela-Pérez et al. 2014 Canada	This study explored the psychological needs of two refugee children.	Qualitative method. A multiple-case research design utilizing storytelling-centered approaches.	Two recent refugee children arriving in Canada. N = 2.	Some children were humiliated and victimized at school and outside school because of their national origin. Peace and tranquility were paramount for the children's narratives. Family members experienced severe distress due to discrimination, isolation, and fear in the host country. Both children struggled to be accepted by and to interact with others.
Vermeyden & Mohamed, 2020 Canada	The study aimed to explore and understand the experiences of Syrian and Iraqi refugees who settled in the Kitchener-Waterloo Region of Ontario, Canada, particularly focusing on their involvement in cultural production activities such as theater and dance.	Case study. Qualitative interviews. Documentary evidence from newspapers and ephemera detailing refugee professional and community theatre and dance productions in the region between 2016 and 2019. Participant observation.	Twenty-eight Syrian and Iraqi refugees who settled in the Kitchener-Waterloo Region. About 10 of these were involved in the artistic productions: Shamrose for Syrian Culture. N = 28.	Participants felt welcomed in Canada due to the way they were received and respected. They stated that Canada is safe for women and children, and they are not worried about walking on the sidewalk or someone hurting the children. Experience of discrimination due to wearing of hijab was reported. Many newcomers actively sought connections with established Canadians through local cultural programs such as English partnership programs. Living in Canada allowed them to engage in cultural activities that were not accessible to them when they were displaced.
Wong & Poon, 2013 Canada	Using storytelling and critical dialogue to engage a group of Hong Kong Chinese immigrant parents in Toronto to interrogate their values and assumptions about homosexuality.	Case study. Storytelling. Data collection methods included: (1) observation of group processes, (2) analysis of project documents including peer parent leaders’ reflective journals, meeting minutes, and newspaper interviews of peer parent leaders, (3) peer parent leaders’ social demographic information, (4) post-training focus groups and individual interviews with peer parent leaders and community partners affiliated with the program, (5) telephone interviews with Chinese parents who participated in the community workshops delivered by the peer parent leaders, (6) community workshop evaluation surveys.	Peer parent leaders who completed the program and participated in post-training focus groups (n = 10). Individual interviews (n = 2). Service providers affiliated with the program who took part in a focus group (n = 4). Community parents who attended workshops delivered by the peer parent leaders and completed a questionnaire (n = 10). All the community parents were Cantonese-speaking immigrants from Hong Kong or Guangzhou, China. All 12 of them (female, n = 11; male, n = 1) were immigrants from Hong Kong and their length of time in Canada ranged from 5 to 25 years, with half of them having been in Canada for less than 10 years. N = 12	The discussion shaped participants’ perspectives about homosexuality and sexual orientation. The majority of participants were confident discussing sexuality with their children. Conversations about sexual orientation enabled parent leaders to talk differently in their work and social lives. Participants’ children experienced a lot of difficulties at school and in trying to adjust to their new environment.

### Stories of Immigration/Settlement Challenges

Four studies (Beauregard et al., 2020; Epp, 2013; Schroeter, 2013; Vermeyden & Mohamed, 2020) described the difficulties that confront newcomers in their quest to integrate into communities within Canada. Through storytelling, immigrants acknowledged the importance of adapting to new communities and milieux and developing their identities as a way of surviving adjustment struggles (e.g., depression, nostalgia, substance abuse, suicidal tendencies, etc.) (Epp, 2013; Vermeyden & Mohamed, 2020). Some refugees recounted obstacles to the preservation of their faith and their languages, namely assimilative pressures and limited opportunities to use their primary dialect, the effects of which were especially pronounced among children and youth given limited original language conversational time with their parents (Vermeyden & Mohamed, 2020). These language barriers coexisted with discrimination as obstacles encountered by immigrants in their neighborhoods (Beauregard et al., 2020; Schroeter, 2013). For instance, language was used as a metric to question the citizenship status of immigrants in Quebec, with some African Canadians harassed because of their inability to communicate in French (Schroeter, 2013), and South Asian immigrant children finding French-language hegemony alienating (Beauregard et al., 2020). Conversely, other immigrants saw the imposition of the English language as tantamount to anti-French cultural erasure, oppression, and colonialism (Beauregard et al., 2020). Storytelling enabled immigrants to articulate and name the stresses and obstacles that prevented them from learning and contributing to their respective communities. In other words, they were able to express, in so many words, adverse mental health circumstances produced by strategies of marginalization.

Five studies (Brushwood Rose & Granger, 2013; Middleton et al., 2021; Schroeter, 2013; Valenzuela-Pérez et al., 2014; Wong & Poon, 2013) documented stories regarding newcomers’ experiences of marginalization and injustice in Canada. Two studies identified migrant social isolation as a by-product of the systemic inequalities that organize Canadian society (Valenzuela-Pérez et al., 2014; Wong & Poon, 2013). These inequalities make it difficult for refugee students and African Canadian youths to adapt to their neighborhoods and reveal a need for more inclusive societies where refugee student–peer interactions can promote good mental health (Schroeter, 2013). This would be an important prescription given the discrimination, misery, isolation, and fear families experience in their transition to Canada (Valenzuela-Pérez et al., 2014). Specifically, Middleton et al. (2021) reported on the way in which immigrants are victimized and humiliated based on their country of origin, citing a participant from Syria who was excluded from playing sports (Middleton et al., 2021). Similar difficulties were explored by some immigrants via storytelling which helped them reconnect with previous experiences, memories, and feelings (Brushwood Rose & Granger, 2013). Finally, some families felt they needed to do more to support the growth of their children, redefine their future, and enhance individual mental health (Wong & Poon, 2013). The studies hitherto described frame storytelling as a restorative practice that names oppressions and, in some instances, conceptualizes states of mental wellbeing that allows participants to envision productive futures.

### Storytelling Creates Safe Spaces

The importance of a sense of safety and inner peace was discussed in nine studies, but varied in terms of how these were addressed and defined through restorative storytelling. Immigrant communities spoke about psychological, emotional, and cultural safety which are critical to their health and wellbeing (Beauregard et al., 2020; Brigham et al., 2018; Chai, 2021; Epp, 2013; Middleton et al., 2021; Perry, 2018; Schroeter, 2013; Valenzuela-Pérez et al., 2014; Vermeyden & Mohamed, 2020). For example, newcomers, when sharing their migration stories, expressed caution using their native language and disclosing their country of origin since these details signify ‘otherness,’ especially if they felt unwelcome and/or had already encountered xenophobic attitudes (Beauregard et al., 2020; Middleton et al., 2021; Vermeyden and Mohamed, 2020). Arts-based interventions helped overcome barriers associated with identity and language through non-judgmental and inclusive sharing of stories (Beauregard et al., 2020; Brigham et al., 2018; Chai, 2021; Middleton et al., 2021; Vermeyden and Mohamed, 2020).

The extent to which participants in the different studies described feeling safe or experiencing elements of safety (e.g., comfort, not feeling judged, feeling welcome), regardless of the ‘type’ of safety, contributed to feelings of tranquility, peace, and stability; these were particularly important in the often turbulent context of migration (Brigham et al., 2018; Chai, 2021; Middleton et al., 2021; Valenzuela-Pérez et al., 2014; Vermeyden & Mohamed, 2020). They also foreground the extent to which safety is an integral aspect of restorative processes and, as such, enable post-turbulence healing. This is more than just a rote description of adversity. For example, in Chai (2021), immigrant women in a Northern Ontario town used photography to present spaces of comfort and discomfort. Shared photos of treacherous winter conditions elicited a sense of vulnerability among immigrant women over the safety of their children (Chai, 2021). Conversely, photos of places associated with comfort, such as libraries, churches, and community centers, fostered internal calm among the participants and provided an opportunity to rest and recuperate from the disorder they experienced (Chai, 2021). Moreover, restorative storytelling processes allowed immigrant children to articulate, organize, and make sense of their migration experiences in ways not afforded by conventional Western approaches (Valenzuela-Pérez et al., 2014). By referring to characters and situations in their stories, they discussed the need for comfort and connection, protection from danger, confronting fear, and the search for familiarity, which connected them to their migratory experiences and invited a better understanding of their psychological needs (Valenzuela-Pérez et al., 2014). These were hopeful and positive counterpoints to adverse experiences that could advance mental wellbeing (Paravati et al., 2022).

Restorative storytelling is a process that narrates and confronts harm in the pursuit of empowerment. Indeed, we discovered that racialized newcomers, forced migrants, and those who were not fluent in the language of the settler majority were able to address stereotypes, reclaim narratives, express frustrations, and create collective knowledge through various forms of artistic or creative expression (Beauregard et al., 2020; Chai, 2021; Epp, 2013; Middleton et al., 2021; Schroeter, 2013; Vermeyden & Mohamed, 2020). Syrian and Iraqi community theatre and dance participants used performative arts to express, navigate, and challenge biased, myopic, and erroneous perceptions of their refugee status and background (Vermeyden & Mohamed, 2020). Similarly, racialized immigrant students and migrant farm workers used the theatrical form known as “theatre of the oppressed” (TO) to unsettle dominant narratives about citizenship and race and explore identity, belonging, and culture in ways that platformed alternative realities of resilience (Schroeter, 2013; Perry, 2018); resilience, of course, being a key pillar of restorative storytelling, broadly conceived (Fitzgerald et al., 2020b; Tenore, 2014). In this regard, four studies went beyond the mere description of trauma and demonstrated how the performing arts enabled cathartic outputs, transformative explorations, and shared meaning making (Beauregard et al., 2020; Perry, 2018; Schroeter, 2013; Vermeyden & Mohamed, 2020). These processes and outcomes are akin to the arts-based narrative therapy model discussed by Epp (2013) which highlighted the importance of community building and narrative healing among immigrants as a means toward self-understanding. To this point, Beauregard et al. (2020) noted that participants “emphasized the importance of the creative process both as a collective and an individual experience” (p. 447), a process that speaks to the exploratory promise of holistic recovery across methods and media, from storytelling and photographs to other shared arts.

### Advancing Social Connectedness and Consciousness

Social connectedness was identified in four studies as social networking or community participation opportunities for immigrants in Canada (Brigham et al., 2018; Hausknecht et al., 2019; Vermeyden & Mohamed, 2020; Wong & Poon, 2013). Sharing stories created awareness, while collaborative learning promoted positive social spaces for people to support one another (Brigham et al., 2018; Hausknecht et al., 2019). For instance, in a study by Hausknecht et al. (2019), participants described knowledge exchange workshops as participatory, encouraging, and valuable because they bring the community together to raise awareness about health and wellbeing. Wong & Poon, (2013) found that during a workshop on family sex education, true dialogue and sharing allowed immigrant participants to express their understanding of sex and sexuality and its implications for their children. Their initial negative perceptions about gender and sexuality changed, and a workshop majority developed confidence discussing these issues with their children (Wong & Poon, 2013). Similarly, the sharing of refugees’ and immigrants’ stories through a multimedia format enhanced connections with their families and others outside the 10-week course that explored digital storytelling (Hausknecht et al., 2019). Brigham et al. (2018) show how participatory photography and community gardening provided immigrant women with opportunities for mutual learning and control over their lives. Finally, research findings from Vermeyden and Mohamed (2020) revealed how local cultural initiatives like English partnership programs allow many newcomers to cultivate social networks with more established Canadians (Vermeyden & Mohamed, 2020). Overall, these restorative storytelling processes heightened awareness of wellbeing, sexuality, and social networking, strengthened intergenerational bonds, and broadened societal connections. Compassion for others (re: sexuality) and narrative reach through technology was also an integral aspect of cultural exchange practices.

Six studies (Beauregard et al., 2020; Chai, 2021; Epp, 2013; Hausknecht et al., 2019; Middleton et al., 2021; Vermeyden & Mohamed, 2020) discussed the role of cultural exchange in promoting mutual learning between immigrants, newcomers, refugees, and new communities. A study by Middleton et al. (2021) showed that immigrant participation in ethnocultural athletics connected landed immigrants and newcomers, which enabled the former to communicate with different people. Another kind of participatory exchange included use of participant-generated photos to humanize research (Chai, 2021; Middleton et al., 2021) in the process of transferring intergenerational knowledge (Epp, 2013; Hausknecht et al., 2019). For example, in a research study by Epp (2013), young people spoke about their eagerness to engage with seniors and discuss generational differences. Moreover, other studies have described how newcomers to Canada desire the support of people from different countries in order to thrive in a new environment (Beauregard et al., 2020; Vermeyden & Mohamed, 2020). According to Beauregard et al. (2020), diverse participants from socioeconomically disadvantaged neighborhoods acknowledged that they require the support of other people to succeed and improve their mental health, while Vermeyden & Mohamed (2020) concluded that the sharing of cultures promotes a sense of belonging regardless of religion and ethnicity. Such exchange also facilitates the development of social consciousness.

Increased social consciousness was a prominent outcome across several articles in this review (Beauregard et al., 2020; Brigham et al., 2018; Brushwood Rose & Granger, 2013; Epp, 2013; Hausknecht et al., 2019; Perry, 2018; Taylor et al., 2022; Vermeyden & Mohamed, 2020; Wong & Poon, 2013). Taylor et al. (2022) identified a theatre-based sex education intervention for newcomer youth in Canada used to promote social change, while Epp (2013) documented the use of arts-based therapy in a Tamil community as a way to build intergenerational alliances (Epp, 2013). Wong & Poon, (2013) describe storytelling as a critical tool for Toronto Chinese immigrants to educate themselves about homophobia (Wong & Poon, 2013). These kinds of narrative processes enabled an exchange of ideas that facilitated social consciousness, with some parents in Wong & Poon, (2013) transforming their prejudices about gender norms and LGBTQ + people in open dialogue with socially aware youth (Wong & Poon, 2013).

In addition, elderly immigrant women engaged in self-reflexivity while composing digital stories that helped them reevaluate their experiences and heal some of their trauma (Hausknecht et al., 2019). Such introspection was also practiced via theatrical processes like dance groups where mixed-performance/mixed-gender national dances from Syria and Iraq were deployed to challenge stereotypes about the Middle East, connect cultures, and develop intercultural identities (Vermeyden & Mohamed, 2020), while in a photo-based study, participants publicly narrated their memories to a wider community via objects and visual metaphors (Brigham et al., 2018) In short, a communion of narrative processes and outcomes among various participants and methods facilitates wellbeing through self-awareness and social consciousness (Beauregard et al., 2020; Brushwood Rose & Granger, 2013; Epp, 2013; Hausknecht et al., 2019; Wong & Poon, 2013; Perry, 2018; Vermeyden & Mohamed, 2020).

### Storytelling Promotes Empowerment and Collective Resilience

Many of the processes associated with restorative storytelling and theatre so far chronicled also lead to outcomes that cultivate mental wellbeing, namely empowerment and collective resilience. Developing resilience and confidence among immigrants is vital to their personal growth as they navigate multiple cultures in Canada. Seven studies acknowledged and explained diverse ways immigrants chart this course through shared stories (Beauregard et al., 2020; Chai, 2021; Epp, 2013; Middleton et al., 2021; Rousseau et al., 2014; Schroeter, 2013; Taylor et al., 2022). One study intimated how drama workshops helped migrant youth transform aspects of past challenges into learning opportunities (Rousseau et al. 2014), while some participants in Beauregard et al. (2020) reported how learning skills such as drawing techniques gave them the courage to learn from their mistakes without judgment and thus take risks.

The above examples of socialized resilience complement the theme of community empowerment. Four studies (Beauregard et al., 2020; Epp, 2013; Middleton et al., 2021; Taylor et al., 2022) presented evidence of how social and community support enabled some immigrants to integrate into their neighborhoods. Epp (2013) explained the importance of community support for immigrants’ resilience in overcoming challenges which have implications for their mental wellbeing. Some immigrants spoke about the community support they received through prayers that helped them develop their faith and resilience (Epp, 2013). Moreover, social learning activities inspired young people to negotiate safer sex and address the shame linked to sex outside of marriage (Taylor et al., 2022). For instance, those trained as peer educators in a newcomer sex education theatre program were empowered to have conversations about sex in immigrant communities where the topic may be taboo (Taylor et al., 2022). Finally, Beauregard et al. (2020) described the role of the *What If?* research project, which explored the processes through which community arts projects in a variety of media promote community resilience. One participant committed to the creative process in ways that made them an empowered agent of change in their community (Beauregard et al., 2020). Participants experienced their engagement in these creative spaces as mending intercommunity tensions and cultivating practices of resilience (Beauregard et al., 2020) that are part and parcel of restorative approaches to storytelling.

**Table 2.** Themes and Sub-Themes Represented by Included Studies.

Themes	Included Studies
Diverse Creative Forms of Storytelling	Brigham et al., 2018; Brushwood Rose & Granger, 2013; Chai, 2021; Epp, 2013; Hausknecht et al., 2019; Middleton et al., 2021; Schroeter, 2013; Taylor et al., 2022
Stories of Immigration/Settlement Challenges	Beauregard et al., 2020; Brushwood Rose & Granger, 2013; Epp, 2013; Middleton et al., 2021; Schroeter, 2013; Valenzuela-Pérez et al., 2014; Vermeyden & Mohamed, 2020; Wong & Poon, 2013
Storytelling Creates Safe Spaces	Beauregard et al., 2020; Brigham et al., 2018; Chai, 2021; Epp, 2013; Middleton et al., 2021; Perry, 2018; Schroeter, 2013; Valenzuela-Pérez et al., 2014; Vermeyden & Mohamed, 2020
Advancing Social Connectedness and Consciousness	Beauregard et al., 2020; Brigham et al., 2018; Chai, 2021; Epp, 2013; Hausknecht et al., 2019; Perry, 2018; Brushwood Rose & Granger, 2013; Taylor et al., 2022; Vermeyden & Mohamed, 2020; Wong & Poon, 2013
Storytelling Promotes Empowerment and Collective Resilience	Beauregard et al., 2020; Chai, 2021; Epp, 2013; Middleton et al., 2021; Rousseau et al., 2014; Schroeter, 2013; Taylor et al., 2022

## Discussion

Increased efforts have been made in Canada on racial justice, community empowerment, and mental health promotion (Hossein, 2021; Moroz et al., 2020; Oliffe et al., 2020), but there must be more done to support people's mental health. Diverse and creative forms of restorative storytelling and community theatre point a way forward with regards to immigrant mental health insofar as they allow immigrants and refugees to: (a) express their migration and settlement experiences; (b) create a communal basis for empowerment and resilience that advances social awareness of immigrants’ mental health; and (c) normalize safe spaces and practices. Restorative storytelling offers people across cultures and identities opportunities to share and resonate with each other's experiences in the contexts of their access to the social determinants of health and wellbeing. When individuals are able to tell their stories in a safe space, they may also be able to strengthen their self-identification and engage in processes of healing as they reflect on their unique and shared experiences of struggle and trauma. In the context of mental health promotion, restorative storytelling and community theatre are *empowerment strategies*; hearing stories of injustices helps participants identify what exactly needs to be *restorative*. As a narrative archaeology of injustices and successes, restorative storytelling and community theatre encourage and shape relationships of trust, consent, mutual recognition, and active listening among participants. In short, they manifest spaces that *humanize* the experiences of participants and, accordingly, develop their resilience and confidence.

To that end, our review centers restorative storytelling as a sense-making practice that enables immigrants to develop a critical social consciousness by reflecting on their experiences. Pioneered by Paulo, critical consciousness is a practice of *mutual empowerment* whereby the marginalized utilize critical dialogue to develop an awareness of how the individual, power relations, and sociocultural practices are fundamentally *changeable* (Chan, 2024; Doll et al., 2023; Freire, 2020). Restorative storytelling and community theatre share an affinity with the practice of critical consciousness so defined by offering a space for immigrants to engage in reflective artistic expression and cultural exchange.

This is demonstrated, despite variations, in all of the studies, which showed evidence of individual and/or interpersonal changes in attitude, understanding, and/or behavior as the result of engaging in the process of arts-based restorative storytelling. This builds on the body of literature that establishes the success of restorative storytelling in stigma reduction and fostering community cohesion, as well as supporting our view that this type of approach leads to mental wellness and restorative justice that is relevant and accessible to marginalized immigrant communities (Budig et al. 2018; Burns et al., 2024; Chioneso et al., 2020; Pichon et al., 2022). Largely, these changes were robust, and some cases extended beyond the parameters of the studies themselves, toward the broader community. Only one study reported negative findings and, even there, these occurred alongside other positive findings (Rousseau et al., 2014).

Storytelling's relational nature emerged as a key aspect of its healing potential, revealing a bidirectional, democratizing dynamic within which the possibility for transformation lies (e.g., opportunities for resistance, co-creation, interpretations of traumatic and/or unjust experiences) (Middleton et al., 2021; Perry, 2018; Vermeyden & Mohamed, 2020; Wong & Poon, 2013). These findings are affirmed by similar studies on the use of collaborative storytelling processes by marginalized communities to facilitate resistance, healing, and self-determination in the wake of racial and historical trauma (Chioneso et al., 2020; Friskie, 2020). Moreover, these processes reveal themselves to be about both discovery *and* recovery, where participants reclaimed the opportunity to critically analyze and reflect on systems of control (Budig et al., 2018; Chioneso et al., 2020; Corntassel et al., 2009; Friskie, 2020; Hawkins & Georgakopolous, 2010; Perry, 2018; Schroeter, 2013).

Restorative storytelling creates an empowering transformative space for oppositional and critical dialogue where participants can confront unexplored or unresolved experiences and challenge dominant narratives, as illustrated in several of the reviewed articles (Perry, 2018; Schroeter, 2013; Wong & Poon, 2013) and bolstered by other research (Budig et al., 2018; Chioneso et al., 2020; Corntassel et al., 2009; Friskie, 2020; Hawkins & Georgakopolous, 2010). Furthermore, theatre emerged as a particularly fertile space for restorative counter-narratives, where play creation encouraged individuals to break away from their usual roles and explore new possibilities (Perry, 2018; Schroeter, 2013; Vermeyden & Mohamed, 2020). This echoed other scholarship on the use of theatre to address racism, social injustice, conflict resolution, and peace-building initiatives (Aguiar, 2020; Bang, 2016; Chamblin et al., 2022; Hawkins & Georgakopolous, 2010). In sharing their stories, impacted communities can make collective sense of their experiences and produce resilient alternative outcomes and solutions (Brigham et al., 2018; Chioneso et al., 2020; Epp, 2013; Friskie, 2020; Perry, 2018; Schroeter, 2013; Vermeyden & Mohamed, 2020; Wong & Poon, 2013).

Our review also advanced previous scholarship showing that restorative storytelling allows for the participation of diverse communities in expressive knowledge creation beyond limitations encountered in more privileged approaches (Corntassel et al., 2009; De Vecchi et al., 2016; Friskie, 2020; Middleton et al., 2021). Two studies included longitudinal components that showed that changes in perspective and behavior among participants were maintained over time and were extended to other community members through subsequent participant-initiated initiatives (Taylor et al., 2022; Wong & Poon, 2013). These findings suggest that restorative storytelling through arts-based approaches offers healing benefits not only to those minoritized communities directly participating in an intervention but also to the broader community, thus showing a greater reach than individualistic approaches. This strength has been described in the scholarship examining the stigma-reducing use of these approaches for HIV and mental illness and in racism and historical trauma (Burns et al., 2024; Chioneso et al., 2020; De Vecchi et al., 2016; Friskie, 2020; Paravati et al., 2022). For example, De Vecchi et al. (2016) examined the use of digital storytelling to improve mental health services by centering the voices of those with lived experience toward educating health care students, practitioners, academics and policymakers on what it is like to live with mental illness.

It is important to emphasize that restorative storytelling is not a panacea for the oppressions faced by marginalized peoples (Schroeter, 2013). Three of the studies we reviewed revealed that empowerment via restorative storytelling is not a given; it requires intentional strategies that challenge dominant discourses and center marginalized storytellers (Perry, 2018; Schroeter, 2013; Wong & Poon, 2013). Schroeter (2013) reported that while Black African-Canadian secondary school students of refugee backgrounds felt “safe enough” to use theatre of the oppressed to challenge their school's administration on some issues, they may not have felt it safe to use it to address race-related discourses. Further research needs to explore how best to use restorative storytelling to address deeply ingrained systemic oppression in ways that are safe for impacted communities.

The sources our review extracted and discussed speak to the archaeological aspects of restorative storytelling, namely how this collaborative process can dig up buried histories to reveal the hardships, barriers, and successes people experienced, the expression of which made people feel humanized, stronger, more resilient, and confident as they reconnected with their culture and/or stood up to unfair treatment. The above findings agree with Dutta et al. (2022) that storytelling empowers individuals to take control of their stories, even helping them transition from perceived victimhood to agency while challenging stereotypes and misconceptions. These shared narratives foster a community of resilience and mutual support to enhance individual and community wellbeing. The promotion of community empowerment and mental health through storytelling highlighted in this review aligns with the outcomes of other recent studies (Chioneso et al., 2020; Dutta et al. 2022). For example, restorative storytelling can help individuals reclaim their humanity by transforming how they perceive themselves and others through the sharing of personal experiences (Chioneso et al., 2020; Dutta et al. 2022). Thus, through storytelling, individuals may present themselves as whole people with full lives, countering stereotypes with personal truth. Storytelling encourages empathy, allowing listeners to understand and connect with different perspectives, thus fostering compassionate communities (Berardi et al., 2020; Fonseka et al., 2021). Through the lens of Critical Race Theory, several studies supported the idea that storytelling is a critical tool for advancing racial justice to avert systemic racial and cultural barriers embedded within society, especially how language policies and racial profiling contribute to discrimination and cultural erasure (Goessling, 2018; Hauber-Özer et al., 2023; Reynolds & Mayweather, 2017).

Our review identified restorative storytelling as an act of empowerment that affirms immigrants’ agency over their narratives, helps them process trauma and build resilience, and contributes to healing and personal growth. Fonseka et al. (2021) affirms this in their claim that storytelling unites communities around common challenges or celebrations to promote solidarity and collective resilience against dominant narratives. This act of reclamation transforms their experiences from passive suffering to active survival and empowerment where, for example, initiatives like theatre-based education not only enhance personal confidence among immigrants but also strengthen community bonds and facilitate deeper integration into new environments (Rousseau et al., 2014; Taylor et al., 2022).

## Limitations

While most of the publications reviewed concluded that restorative storytelling provides an important space for personal and community catharsis, empathetic understanding, and resistance, some important limitations were noted. Our study was limited to peer-reviewed studies in Canada, making it difficult for us to draw important lessons on restorative storytelling and immigrant mental wellbeing from other geographical regions. We also excluded peer-reviewed studies on restorative storytelling and immigrants’ mental wellbeing in Canada that were not published in the English language. Since our review did not account for gray literature and government documents in Canada, it was difficult for us to draw conclusions on government programs and related activities on restorative storytelling within the timeframe. Furthermore, it is important to note that stigma of mental illness is a key barrier to help seeking among racialized migrants and refugees (Mohammadifirouzeh et al., 2023). Thus, our review focusing on restorative storytelling for racial justice and empowerment only offers a partial picture. The reviewed articles did not address the conditions required to enhance mental health-related help-seeking behaviors among migrants and refugees; they also did not capture the differences in help seeking across different generations. Studies of restorative storytelling for mental health promotion need to consider stigma reduction and acculturation as potential preceding conditions for participation and include it in the intervention design.

## Conclusion

The current review analyses recent empirical studies on restorative storytelling and community theatre as a tool to promote the community empowerment and mental wellbeing of immigrants in Canada. Our findings largely show that immigrants are confronted with several challenges in their attempts to settle in Canada which negatively impact their mental health. It is evident that some immigrants struggle to preserve their faith and languages given the short expectation to learn the English language and few opportunities to regularly use their first languages. However, through restorative storytelling, some immigrants have created a positive social space to support one another via collaborative learning and social networking. The exchange of culture through storytelling also fosters diversity and promotes social inclusion and a sense of belongingness among immigrants regardless of their religion and ethnicity. This social connectedness allows people to explore diversity through distinct activities and dialogue to inspire associations between members of varied age groups. It is worth noting that, despite all the different strategies deployed in restorative storytelling, broadly conceived, no single modality emerged strongly as being more or less effective than the other. Also, we cannot conclude that a particular demographic emerged as better or worse suited to such interventions. Only one study reported negative findings and a difference between sub-groups of immigrant participants, noting that first-generation immigrant students exposed to their theatre intervention responded positively by showing improvements in self-reported emotional and behavioral symptoms, whereas second-generation immigrant students’ self-reports worsened due to institutional barriers (Rousseau et al., 2014)*.* Given the increasing mental health disparities in immigrant and racialized communities in Canada, efforts toward improving the mental wellbeing of immigrants through restorative storytelling warrant further investigations.

## Data Availability

Data sharing does not apply to this article as no new data were created or analyzed in this study. All the articles used in the analysis can be retrieved from public libraries with the necessary DOI.
